# Indicators of verbal creative thinking: results of a Delphi panel

**DOI:** 10.3389/fpsyg.2024.1397861

**Published:** 2024-07-15

**Authors:** Olivia López Martínez, Antonio José Lorca Garrido, María Isabel de Vicente-Yagüe Jara

**Affiliations:** ^1^Department of Developmental and Educational Psychology, University of Murcia, Murcia, Spain; ^2^Department of Didactics of Language and Literature, University of Murcia, Murcia, Spain

**Keywords:** verbal creativity, children, fluency, flexibility, originality

## Abstract

**Introduction:**

Creativity is a fundamental competence that manifests itself in various domains of knowledge, including verbal creativity. The main aim of this study was to identify indicators of verbal creativity for the assessment of three writing tasks.

**Methods:**

Sixteen multidisciplinary and international creativity experts participated in a two-stage Delphi panel. The administered questionnaire asked about the measurement or non-measurement of eight indicators of verbal creative thinking in three tasks: problem posing, creative idea generation, and idea improvement. Originality is the most important indicator of creativity. The indicators identified in the first task were fluency, flexibility, originality, elaboration, and sensitivity to problems. The second task measures flexibility, originality, elaboration, opacity, and dynamic integration. In the third task, fluency, flexibility, originality, elaboration, dynamic integration, and refinement of ideas are considered.

**Results:**

The results of this study are key to progress in the field of measuring verbal creative thinking.

**Discussion:**

The identification of indicators of the construct called verbal creativity allows the determination of its components in order to be able to estimate the creative potential in this specific domain.

## 1 Introduction

Creativity is an element that requires potential originality and effectiveness in a dynamic context (Corazza et al., 2022), which has become increasingly important in recent years. Creative thinking not only underpins some of society’s most important innovations but is also a universal and democratic phenomenon (OECD, 2020). Currently, the concern to assess creativity has led to its inclusion by the OECD in the Program for International Student Assessment (PISA). Creative thinking has gained the status of a competency that helps individuals achieve better outcomes in more difficult and challenging contexts (OECD, 2020).

Traditionally, creativity measurement has been approached from a domain-general perspective (Guilford, 1950; Torrance, 1974). However, the trend has changed nowadays, as most authors (Kaufman et al., 2017; Baer, 2019; Zyga et al., 2022) understand creativity from the point of view of domain-specificity, and therefore, stating that someone is creative without alluding to the domain of knowledge in which he or she is creative would be an incorrect statement.

Many authors (Amabile, 2018; Lorca Garrido et al., 2021; Krieger-Redwood et al., 2023) agree that verbal creativity is one of the specific domains of creativity, because they do not find correlations between this specific domain and the rest of the specific domains of creativity. Therefore, the measurement of verbal creativity is the domain of interest of the present research, and it is defined as the value of divergent thinking in solving problems with verbal content (Portnova et al., 2020). For their part, López-Martínez et al. (2018) understand verbal creativity as a linguistic act composed of the activation of creative thinking and a process of written reflection that allows the elaboration of a narrative with textual harmony, metaphors, originality and imagination.

The domain in which creativity is manifested is an important moderator of the relationships between creativity and factors such as personality or communication. For this reason, more efforts are needed to develop an instrument capable of assessing creativity, understood from the point of view of the specificity of the domain, since its conceptualization and explanatory theories differ from most of the validated instruments.

Furthermore, it should be noted that creativity, as a psychological construct, is not directly observable, so in the various existing tests for measuring creativity, a set of observable indicators has already been delimited, which allowed to accurately determine the level of creative thinking manifested by each individual (Runco and Acar, 2019). In this sense, Guilford (1950) already established that the indicators that structure creative thinking are fluency, flexibility, originality, redefinition, penetration, and elaboration. Nevertheless, in tests of divergent thinking, only the scores of fluency, flexibility, originality, and elaboration are usually used to estimate the creative potential of individuals (Runco and Acar, 2019; Corbalán, 2022).

First, fluency is considered to be a characteristic of creative minds that consists of the spontaneous flow of ideas and images (Kasirer and Mashal, 2018). Therefore, the minds of creative individuals have a greater ability to generate multiple ideas when approaching a task. Second, flexibility is characterized by the categorization of ideas into different categories, which is a sign of the variety and diversity of ideas contributed by an individual (Acar et al., 2019). Therefore, ideas contributed to different categories are valued more from a creative perspective than those belonging to the same category from an operational perspective (Guilford, 1956).

Third, originality is the most important indicator of creativity. In fact, creativity is understood as intentional originality (Pichot et al., 2022). This indicator refers to the uniqueness and unusualness of the given ideas compared to those of other participants (Forthmann et al., 2021). Currently, one of the methods used to objectively measure originality is latent semantic analysis in text mining, as it provides a computerized originality score based on the cosine of the angle formed between the stimulus word and the word given by the individual (Beaty and Johnson, 2021; Dumas et al., 2021).

Fourthly, elaboration refers to the details that are pointed out when stating an idea, which embellish the idea (Acar et al., 2019). This ability also refers to the degree of difficulty achieved by the individual in defining conceptual structures (Guilford and Hoepfner, 1971). One of the most widely used methods for interpreting elaboration is the unweighted word count, where elaboration is understood as the number of words in each of the verbal responses provided by the subject in a given verbal divergent thinking task (Maio et al., 2020).

Guilford (1968) significantly expanded the above indicators and introduced problem sensitivity as a person’s ability to identify problems that not everyone perceives, an indicator also considered by Violant (2006) and Romo et al. (2016). Recently, the OECD (2020) introduced a new indicator to measure creative thinking, which is the refinement of ideas, which consists in the evaluation and improvement of an already completed task, with the aim of facilitating the development of a more appropriate and original response than the one initially posed.

In this sense, Desrosiers (1978) argues that in order to include the divergent aspects of communicative written expression, it is necessary to use figurative language, which is constituted by imagination, originality, flexibility, dynamic integration and a certain degree of opacity. The author alludes to the divergent thinking in this field from the use of an embellished language as it is usually done in poetry. In fact, he raises the indicators of opacity for the use of a language in which the relationship between signifier and signified is diluted and dynamic integration as the factor that alludes to the uniqueness of a text, focusing attention on linguistic aspects.

Currently, tests of divergent thinking are based on creativity from a general point of view, as evidenced by the fact that the indicators of creativity they evaluate are fluency, flexibility, and originality, which are general indicators of creativity. These dimensions are evaluated in the Creative Imagination Test for Children (PIC-N) by Artola et al. (2004) and in the Torrance Test Creative Thinking (TTCT) by Torrance (1974), even in the tasks of these tests, which are considered verbal, the elaboration is left aside, probably because it implies an additional difficulty for its evaluation. Other tests, such as the CREA (Creative Intelligence test) by Corbalán et al. (2003), only measure fluency based on the number of questions elaborated by the students. Likewise, all the tests are aimed at ideational productivity, which is a limitation of the tests because of the absence of the critical and evaluative dimension of ideas (Runco, 2008), so they lack a task in which the evaluation and improvement of ideas is considered.

Later, the Verbal Creativity Test (PCV in Spanish or VCT in English) (López-Martínez et al., 2018) emerged to try to provide an answer to the problem of the lack of consideration of verbal creativity in research on creative thinking. However, its main objection is the subjectivity of its indicators and the small sample with which it has been validated.

In addition, with respect to the context and age to which the creativity tests are addressed, the current research requires tests intended for a psychoeducational context of application and for a population of primary school students who use Spanish as their native language. Also, in order to respond to American Educational Research Association, American Psychological Association, and National Council on Measurement in Education (2018), the objectives of the creativity test to be designed are to identify talents in the field of linguistics, to guide the educational attention of students according to their characteristics and to assess the specific verbal domain of creativity, as well as to determine the starting point of an intervention and monitor their progress. Furthermore, in order to overcome the inconveniences of other creativity tests, the test to be designed must be aimed at assessing verbal creative thinking based on the classical indicators of creativity aimed at narrative and linguistic aspects, as well as others related to the verbal component.

In this test, the tasks must consider the generation of ideas, the elaboration of creative ideas, and the improvement and evaluation of ideas (OECD, 2020; Matheson et al., 2023), as well as considering problem posing as an important starting point of creativity (Abdulla Alabbasi et al., 2021), in which questions are the backbone of creative thinking (Corbalán et al., 2003). Likewise, it should be taken into account that in a psychometric test to assess verbal creativity, texts must play a fundamental role (López-Martínez et al., 2018), since they can be both the stimulus for a creative activity and the expression of the creative performance in the form of a creative product.

Regarding the universal definition of the construct, the American Educational Research Association, American Psychological Association, and National Council on Measurement in Education (2018) indicated the need to provide an operational, semantic, and syntactic definition of the construct. In this sense, the definitions of the construct proposed in the present study are given below. First, verbal creative thinking operationally can be understood as the observable quantity of the presence of the following indicators of creativity in its verbal domain, which are fluency, sensitivity to problems, flexibility, originality, elaboration, opacity, dynamic integration, and refinement of ideas. Second, the semantic definition of verbal creative thinking is characterized by the following facets, which are problem posing, creative idea generation, and evaluation and improvement of ideas within the verbal domain of creativity from different stimuli in text form. Third, verbal creative thinking in syntactic form is a construct that shows relationships with reading comprehension, intelligence, personality, and emotions.

Finally, following Muñiz and Fonseca-Pedrero (2019), the characteristics of the tasks that make up the test should be specified, that is, the number, length, content, order, and response format. With regard to the tasks, it should be indicated that they correspond to the type called essay, since, due to the nature of the construct of verbal creative thinking, they are those that allow the individual to express themselves freely in order to show their creative capacity (Muñiz, 2018). It should be mentioned that their evaluation involves greater difficulty than the other types of tasks, since the subject is not limited by response alternatives, in addition to the greater effort that must be made to measure verbal creative thinking.

However, it is more correct to refer to the term as reagents or stimuli, instructions, indicators of verbal creative thinking, and examples that are presented in the context of a task or game. In total, there are three tasks with the following order and content: (a) Task 1: problem posing; (b) Task 2: generating creative ideas; (c) Task 3: evaluating and improving ideas.

In addition, the following eight observable indicators of verbal creative thinking are used for evaluation: fluency, flexibility, originality, elaboration, sensitivity to problems, opacity, dynamic integration, and refinement of ideas. The identification of these indicators in each of the tasks could be carried out by consulting the experts who are the most knowledgeable on the subject, by using the Delphi method as the appropriate methodology.

The Delphi method is based on a communicative process in which a group of experts gives an answer to a research problem through an iterative process (López-Gómez, 2018). This process takes place anonymously, iterative questionnaire-results-questionnaire, which leads to feedback from the experts so that they can reach the highest possible consensus among them to achieve the proposed objective (Plans and León, 2003).

The value of this technique for the development creativity is that it makes it possible to coordinate the different preferences, currents, trends, and lines contributed by experts in the field. In this sense, it was decided to use the Delphi method, as it was considered the most appropriate to achieve the objective of this study: to determine which indicators of verbal creativity could be measured in each of the tasks.

## 2 Materials and methods

A Delphi method was used to reach a consensus among creativity experts in order to determine the indicators of verbal creative thinking.

### 2.1 Participants

For the selection of the participants, a non-probabilistic purposive sampling was used (Jorrín et al., 2021), since the experts who met the following criteria were selected: availability and interest in participating in this study, at least 5 years of experience in the study of creativity and research activity, accredited by publications of impact (JCR or SJR).

Based on these criteria, 40 experts were invited to participate in a Delphi panel on indicators of verbal creative thinking. Twenty agreed to participate, but only 16 completed the two phases of the Delphi method, resulting in an attrition rate of 20% (*n* = 4). Despite this, the ideal number of experts to participate in the Delphi method was met (López-Gómez, 2018). Table 1 shows the profile of the experts who participated in the present study.

**Table 1:** Expert profiles.

Characteristic		*n*	%
Gender	Masculine	6	37.5
	Feminine	10	62.5
Home University	University of Murcia, Spain	11	68.75
	University Miguel Hernández, Spain	1	6.25
	National University of Rio Cuarto, Argentina	2	12.5
	National University of Jujuy, Argentina	1	6.25
	Drake University, Iowa, USA	1	6.25
Professional category	University Professor	1	6.25
	University Associate Professor	8	50
	University Adjunct Professor	7	43.75
Area of expertise	Educational Psychology	6	37.5
	Language Didactics	1	6.25
	Basic Psychology	1	6.25
	Personality, Psychological Assessment and Treatment	3	18.75
	Psychometry	1	6.25
	Didactics of Artistic Expression	1	6.25
	Philosophy of Education	1	6.25
	Methodology of Behavioral Sciences	1	6.25
	Industrial Psychology	1	6.25
Teaching and research experience	5–15 years	4	25
	15–25 years	5	31.25
	More than 25 years	7	43.75

This Delphi panel included experts from nine different fields of knowledge, with 25% of the sample being foreigners. In addition, the percentage of women (62.5%) and the fact that 43.75% of the experts had more than 25 years of experience stood out.

To determine the quality of the expert panel, the Competence (*K*) index (Cabero and Barroso, 2013) was calculated, which is the sum of the knowledge coefficient (Kc) and the argumentation coefficient (Ka) divided by 2. Both the Kc (*M* = 0.84; *SD* = 0.09) and Ka (*M* = 0.81; *SD* = 0.18) scores were high, which means that *K* is high (*M* = 0.825; *SD* = 0.1). With an Expert Competence score above 0.8, the Delphi panel is considered to be of high quality for addressing the research problem (Table 2).

**Table 2:** Quality of the expert panel.

Expert index	*M*	*SD*
Knowledge coefficient	0.84	0.09
Argumentation coefficient	0.81	0.18
Competence index	0.825	0.1

### 2.2 Instruments

To collect information from the participants, we used an *ad hoc* questionnaire developed by our research group. This questionnaire was divided into four parts. The first part was used to collect socio-demographic data that determined the characteristics of the Delphi panel. The rest of the questionnaire was composed of 24 items corresponding to the indicators to be measured in each of task. The latter included a dichotomous scale in which the experts had to indicate whether they would or would not measure these indicators in Game 1 of problem posing, in Game 2 of generating creative ideas, and in Game 3 of improving ideas. The indicators were: fluency, flexibility, originality, elaboration, sensitivity to problems, opacity, dynamic integration, and refinement of ideas.

### 2.3 Procedure

In the first phase of the Delphi method, the experts received an invitation by e-mail together with the digital version of the questionnaire and were given a period of time to complete it. Once all the questionnaires had been returned, the results of this first phase were grouped into percentages and these results, together with the questionnaire, were sent to carry out the second phase of the Delphi method. In this phase, the experts considered the responses from phase 1 to answer the questionnaire, with the aim of reaching a minimum consensus of 80% for each of the indicators. These experts could not interact with each other, and their anonymity was guaranteed in both phase 1 and phase 2.

### 2.4 Data analysis

Data analysis was performed using IBM SPSS Statistics version 28.0.1. Descriptive statistics and frequencies were used to describe the panel of experts. For each indicator, participants responded with the following options: 1 = Yes and 2 = No. This dichotomous scale was used to reduce ambiguity, for its objectivity and clarity, and to allow the experts to focus more on the relevance of the indicator. Once the degree of agreement at the percentage level among the experts is known through the use of frequencies, it becomes necessary to check whether the agreements reached are statistically significant. Frequencies were used to determine the percentages of expert responses. The Fleiss kappa test was also used to determine the coefficient of concordance between the experts’ responses in each of the games and phases of the Delphi method. To interpret the results of Fleiss’ kappa coefficient, the Landis and Koch (1977) scale was used to determine the strength of agreement of the experts. This scale has the following values: poor (0), slight (0.1–0.2), acceptable (0.21–0.4), moderate (0.41–0.6), substantial (0.61–0.8), and near perfect (0.81–1). Participants’ responses were analyzed in both rounds because they could keep or change their responses.

## 3 Results

Table 3 shows the indicators that the experts considered relevant for measuring the construct of verbal creative thinking in each of the three games in phase 1 of the Delphi method. For the indicators of the first game in phase 1, the experts considered that indicators such as fluency (100%), flexibility (81.25%), and sensitivity to problems (93.75%) should be measured, while refinement of ideas (81.25%) should not be measured. The overall agreement achieved by the experts in Game 1 was acceptable and statistically significant (Kappa = 0.35, *Z* = 10.853, *p* < 0.01) with a 95% confidence interval (0.287–0.414).

**Table 3:** Delphi method phase 1 results.

Game	Indicators	Yes (%)	No (%)	Fleiss’ kappa (*p*)
Game 1	Fluency	16 (100%)	0 (0%)	0.35 (0.000)
	Flexibility	13 (81.25%)	3 (18.75%)	
	Originality	11 (68.75%)	5 (31.25%)	
	Elaboration	11 (68.75%)	5 (31.25%)	
	Sensitivity to problems	15 (93.75%)	1 (6.25%)	
	Opacity	4 (25%)	12 (75%)	
	Dynamic integration	4 (25%)	12 (75%)	
	Refinement of ideas	3 (18.75%)	13 (81.25%)	
Game 2	Fluency	7 (43.75%)	9 (56.25%)	0.178 (0.001)
	Flexibility	12 (75%)	4 (25%)	
	Originality	16 (100%)	0 (0%)	
	Elaboration	13 (81.25%)	3 (18.75%)	
	Sensitivity to problems	5 (31.25%)	11 (68.75%)	
	Opacity	10 (62.5%)	6 (37.5%)	
	Dynamic integration	14 (87.5%)	2 (12.5%)	
	Refinement of ideas	7 (43.75%)	9 (56.25%)	
Game 3	Fluency	9 (56.25%)	7 (43.75%)	0.114 (0.001)
	Flexibility	12 (75%)	4 (25%)	
	Originality	14 (87.5%)	2 (12.5%)	
	Elaboration	13 (81.25%)	3 (18.75%)	
	Sensitivity to problems	7 (43.75%)	9 (56.25%)	
	Opacity	9 (56.25%)	7 (43.75%)	
	Dynamic integration	14 (87.5%)	2 (12.5%)	
	Refinement of ideas	16 (100%)	0 (0%)	

Global agreement on Delphi panel phase 1 (Fleiss’ kappa = 0.232, *p* < 0.001).

For the second set of indicators, the experts determined that originality (100%), elaboration (81.25%), and dynamic integration (87.5%) should be evaluated. The overall agreement among the experts was lower because it was manifested at a low level, although statistically significant (Kappa = 0.178, *Z* = 5.517, *p* < 0.01) with a 95% confidence interval (0.115–0.241).

In the third game, it was agreed to measure the indicators of originality (87.5%), elaboration (81.25%), dynamic integration (87.5%), and refinement of ideas (100%). Fleiss’ kappa statistic shows a slight and statistically significant agreement among the experts in game 3 (Kappa = 0.114, *Z* = 3.518, *p* < 0.01) with a 95% confidence interval (0.05–0.177), which is the lowest of the three games in this first phase.

Considering all the games simultaneously, that is, all the answers given by the experts in Phase 1, an acceptable and statistically significant level of global agreement was obtained (Kappa = 0.232, *Z* = 12.444, *p* < 0.01) with a 95% confidence interval (0.195–0.268). Although statistically significant agreements were obtained in Fleiss’ kappa statistic, these agreements are low and acceptable, which means that they are not sufficient to conclude the Delphi method. For this reason, it was necessary to conduct a second round of Delphi to achieve a higher level of agreement.

In phase 2 of the Delphi method, the experts were able to consider the results of phase 1 in order to modify or maintain their answers. In this second phase, higher levels of agreement were expressed for each of the games, as shown in Table 4. 100% of the experts indicated that the indicators of fluency, flexibility and sensitivity to problems should be measured in Game 1, just as 100% of the experts indicated that the indicators of opacity and dynamic integration should not be measured. In Game 1, the overall agreement among the experts was almost perfect and statistically significant (Kappa = 0.821, *Z* = 25.425, *p* < 0.01) with a 95% confidence interval (0.757–0.884), meaning that it was the highest level of agreement among all the games.

**Table 4:** Delphi method phase 2 results.

Game	Indicators	Yes (%)	No (%)	Fleiss’ kappa (*p*)
Game 1	Fluency	16 (100%)	0 (0%)	0.821 (0.000)
	Flexibility	16 (100%)	0 (0%)	
	Originality	15 (93.75%)	1 (6.25%)	
	Elaboration	13 (81.25%)	3 (18.75%)	
	Sensitivity to problems	16 (100%)	0 (0%)	
	Opacity	0 (0%)	16 (100%)	
	Dynamic integration	0 (0%)	16 (100%)	
	Refinement of ideas	2 (12.5%)	14 (87.5%)	
Game 2	Fluency	2 (12.5%)	14 (87.5%)	0.688 (0.000)
	Flexibility	14 (87.5%)	2 (12.5%)	
	Originality	16 (100%)	0 (0%)	
	Elaboration	16 (100%)	0 (0%)	
	Sensitivity to problems	1 (6.25%)	15 (93.75%)	
	Opacity	14 (87.5%)	2 (12.5%)	
	Dynamic integration	16 (100%)	0 (0%)	
	Refinement of ideas	3 (18.75%)	13 (81.25%)	
Game 3	Fluency	13 (81.25%)	3 (18.75%)	0.628 (0.000)
	Flexibility	14 (87.5%)	2 (12.5%)	
	Originality	16 (100%)	0 (0%)	
	Elaboration	16 (100%)	0 (0%)	
	Sensitivity to problems	2 (12.5%)	14 (87.5%)	
	Opacity	3 (18.75%)	13 (81.25%)	
	Dynamic integration	16 (100%)	0 (0%)	
	Refinement of ideas	16 (100%)	0 (0%)	

Global agreement on Delphi panel phase 2 (Fleiss’ kappa = 0.723, *p* < 0.001).

In Game 2, the experts showed 100% agreement in judging originality, elaboration, and dynamic integration. Fleiss’ kappa statistic shows considerable and statistically significant agreement among the experts in Game 2 (Kappa = 0.688, *Z* = 21.311, *p* < 0.01) with a 95% confidence interval (0.625–0.751).

In addition, 100% inter-rater agreement was achieved in Game 3 for the measures of originality, elaboration, dynamic integration, and refinement of ideas. The overall agreement reached by the experts in Game 3 was considerable and statistically significant (Kappa = 0.628, *Z* = 19.451, *p* < 0.01) with a 95% confidence interval (0.565–0.691), being the game with the lowest level of agreement of the three games, as was the case in the first phase of the Delphi method.

In the whole phase 2 of the Delphi method, a considerable and statistically significant level of global agreement was obtained (Kappa = 0.723, *Z* = 38.824, *p* < 0.01) with a confidence interval of 95% (0.687–0.76). The percentage agreement of 80% was obtained for each of the indicators of verbal creative thinking, which is considered key for the measurement or not of each of them in the different games. Likewise, Fleiss’ kappa statistic showed considerable and almost perfect degrees of agreement in this second phase, significantly increasing the levels of agreement that had occurred in the first phase. Therefore, the Delphi method was stopped in this second phase.

## 4 Discussion

The results obtained show that the experts consulted consider that the main indicator of verbal creative thinking is originality (Forthmann et al., 2021; Pichot et al., 2022), understanding that the original ideas contributed by the subjects in the proposed tasks are distant in meaning from the task stimuli (Beaty and Johnson, 2021; Dumas et al., 2021).

To originality are added elaboration and flexibility, since their measurement is considered in the three games, appreciating a clear tendency of the experts for the classic indicators of creativity. Also considered, although to a lesser extent since it is only evaluated in three games, is fluency, which is another of the classic indicators on which estimates of the creative thinking of individuals are based (Runco and Acar, 2019; Corbalán, 2022). In fact, most creativity tests usually measure fluency, flexibility, and originality in their verbal tasks (Torrance, 1974; Corbalán et al., 2003; Artola et al., 2004; López-Martínez, et al., 2018). However, the indicator that is not usually measured in verbal tasks is elaboration, which is usually considered in figurative tasks.

The indicators used to assess verbal creative thinking in a problem-solving task were fluency, flexibility, originality, elaboration, and sensitivity to problems. The indicator that was added to those already described was sensitivity to problems (Guilford, 1968). This indicator is considered to be the starting point of creative thinking, since problem posing, and problem finding are responsible for the emergence of creativity.

In addition, flexibility, originality, elaboration, opacity, and dynamic integration are the key indicators to be measured in a creative idea generation task in the evaluation of the construct. This type of task is usually carried out in creativity tests, although in this case there is the specificity of including opacity, in which non-literal language is sought, and dynamic integration, which focuses on the unity of the elaborated text (Desrosiers, 1978; López-Martínez, et al., 2018). The incorporation of these indicators implies the transition from general creativity to verbal creative thinking.

In Game 3 regarding the improvement of ideas, the measurement of fluency, flexibility, originality, elaboration, dynamic integration, and refinement of ideas is considered crucial. In addition to the indicators described above, the refinement of ideas is a key indicator in a task where the goal is to improve a given product with the aim of making it suitable for solving the task at hand and original compared to the rest of the individuals (OECD, 2020; Matheson et al., 2023). This task is the last step of verbal creative thinking. It should be mentioned that usually only tasks of idea generation are given to the detriment of tasks of problem solving and improvement of ideas.

In conclusion, it should be noted that the detection of verbal creative thinking is key to its development in the school context. The tools used to measure the verbal area of creativity are not sufficient because they do not consider all the indicators identified in this study that make it possible to measure this thinking (Guilford, 1968; Desrosiers, 1978; Kasirer and Mashal, 2018; Acar et al., 2019; Beaty and Johnson, 2021; Forthmann et al., 2021). For this reason, this information is valuable for the development of a tool that assesses verbal creative thinking in an appropriate manner, using texts that have key relevance in this verbal domain.

Measuring it is a step forward in this facet, as creativity is one of the 21st century competencies that allows society to adapt and solve problems (OECD, 2020; Corazza et al., 2022). Teachers play a fundamental role in its improvement, as they oversee the implementation of educational practices that allow its progress through creative intervention programs and the development of a creative curriculum. They are also key to identifying talent in the area of knowledge most closely related to language, writing and reading.

The main limitation of this study was the choice of creativity indicators. This did not allow the experts to consider indicators other than those previously established for measurement in each of the games. However, the selection of indicators was made after a thorough review of the scientific literature. The intention of this selection was that the experts would consider both traditional indicators of creativity, which are widely known, as well as specific indicators of verbal creativity, which have not been considered as much in the evaluation of this thought in the various tests of creativity. In fact, it was the combination of both in the different tasks that made it possible to measure the construct called verbal creative thinking.

Once the indicators of Verbal Creative Thinking have been identified in the present study, the main line of future research is to develop a test to estimate the verbal creative potential, which includes the content validity of the test and the development of a pilot study with the intended participants of the test, individuals in primary education. Also, this test will serve not only to work on aspects of reading comprehension but also to prevent language development disorders.

## Data availability statement

The raw data supporting the conclusions of this article will be made available by the authors, without undue reservation.

## Ethics statement

The studies involving humans were approved by Comisión de Ética de Investigación – Universidad de Murcia. The studies were conducted in accordance with the local legislation and institutional requirements. The participants provided their written informed consent to participate in this study.

## Author contributions

OL: Writing – original draft, Writing – review & editing. AL: Writing – original draft, Writing – review & editing. MV-Y: Writing – original draft, Writing – review & editing.
